# SiGNet: A signaling network data simulator to enable signaling network inference

**DOI:** 10.1371/journal.pone.0177701

**Published:** 2017-05-17

**Authors:** Elizabeth A. Coker, Costas Mitsopoulos, Paul Workman, Bissan Al-Lazikani

**Affiliations:** Cancer Research UK Cancer Therapeutics Unit, The Institute of Cancer Research, London, United Kingdom; Friedrich-Alexander-Universitat Erlangen-Nurnberg, GERMANY

## Abstract

Network models are widely used to describe complex signaling systems. Cellular wiring varies in different cellular contexts and numerous inference techniques have been developed to infer the structure of a network from experimental data of the network’s behavior. To objectively identify which inference strategy is best suited to a specific network, a gold standard network and dataset are required. However, suitable datasets for benchmarking are difficult to find. Numerous tools exist that can simulate data for transcriptional networks, but these are of limited use for the study of signaling networks. Here, we describe SiGNet (Signal Generator for Networks): a Cytoscape app that simulates experimental data for a signaling network of known structure. SiGNet has been developed and tested against published experimental data, incorporating information on network architecture, and the directionality and strength of interactions to create biological data in silico. SiGNet is the first tool to simulate biological signaling data, enabling an accurate and systematic assessment of inference strategies. SiGNet can also be used to produce preliminary models of key biological pathways following perturbation.

## Introduction

The application of networks and graph theory to biological systems is becoming increasingly important, particularly for understanding disease biology and drug action, and for selecting appropriate biomarkers or therapeutic interventions [1]. Since the human interactome is not fully mapped and cellular wiring varies in different cellular contexts, the structure of cellular networks should ideally be inferred from experimental data [2]. Inference techniques can be used to identify causal links between the levels of different biological entities, for example whether protein X activates protein Y [3, 4], or to identify the structures of gene regulatory networks [5, 6]. There are a plethora of inference strategies available, including those based on mutual information [7, 8], Bayesian [9] and information-theoretic approaches [10]. Therefore, some means of objectively determining the best-performing inference strategy is needed to optimize the utility of the inferred network and enable its application in translational research.

To develop the best inference approaches, the research community needs data from a network of known structure. This would enable quantitative validation of alternative methods. Unfortunately, real, large-scale and times series biological data for such ‘gold standard’ networks is extremely difficult to find. Numerous datasets have been made public to facilitate machine learning in other areas [11], but these are of limited relevance to biological signaling networks. Traditionally, perturbations in the levels of signaling proteins resulting from the application of targeted drugs or siRNA are demonstrated by Western blotting. However, this technique provides only semi-quantitative data unless appropriate calibration procedures have been used and described [12]. Although journals are placing increasing emphasis on the quantification of Western blots [13], it is still rare to find quantitative protein data for a complete signaling network of known structure, especially for time series. In addition to this, Western blotting only indicates the abundance of the protein of interest, which does not necessarily correlate with protein activity. Almost no public, longitudinal experimental data exist for signaling networks: rare exceptions include the LINCS Project [14] which has yielded unpublished, downloadable data of ERK protein dynamics in a single cell line in response to four small molecule inhibitors of ErbB kinase. In contrast, the task of developing inference techniques for transcriptional networks is made easier by the fact that transcript levels are often quantified using microarrays, raw data from which are freely available through repositories such as ArrayExpress [15] and GEO [16]. As a result, several tools exist for generating transcriptional networks and datasets in silico, for example GeneNetWeaver [17] and GRENDEL [18]. No such tools exist for protein signaling networks.

Transcriptional networks and signaling networks are, by definition, different in structure and timescale. Signaling networks must respond to stimuli rapidly [19], whereas transcriptional networks may need to produce sustained patterns of activity over time [20]. In addition, transcription is often controlled by a relatively small number of transcription factors acting on many targets, whereas signaling cascades and pathways typically form a more linear network with additional feedback loops etc. This means that a transcriptional network will have a different architecture to that of a signaling network. For these reasons, data generated from simulations of transcriptional networks is unsuitable for benchmarking a protein signaling study.

To align simulations with real biological systems, networks may need to be constructed with a significant bias towards a particular structure or motif (e.g. a signaling cascade such as the MAPK pathway, or a pathway with a high degree of cross-talk, such as the PI3K/mTOR pathway). Therefore, there is a need for a bespoke tool that can generate simulated experimental data for a signaling network defined by the user. The lack of suitable benchmarks has been a challenge since inference strategies were first applied to signaling networks. In 2005, the structure of a small network of 11 proteins was inferred from experimental protein phosphorylation data [21]. The models generated were scored according to whether their inferred edges (interactions between proteins/nodes that had been computationally inferred) matched the edges seen in a ‘conventionally accepted’ synopsis of signaling interactions between the proteins. This benchmarking strategy has two major issues. Firstly, there is no universally-accepted definition of a biological signaling pathway, and the human interactome has yet to be fully mapped [22]. Secondly, the ‘gold standard’ used in this study is an amalgamation of mammalian interactions, yet the authors inferred from it a human primary T cell network. This highlights the need for a tool to simulate bespoke benchmarking data for the signaling network being studied.

**Fig 1:**
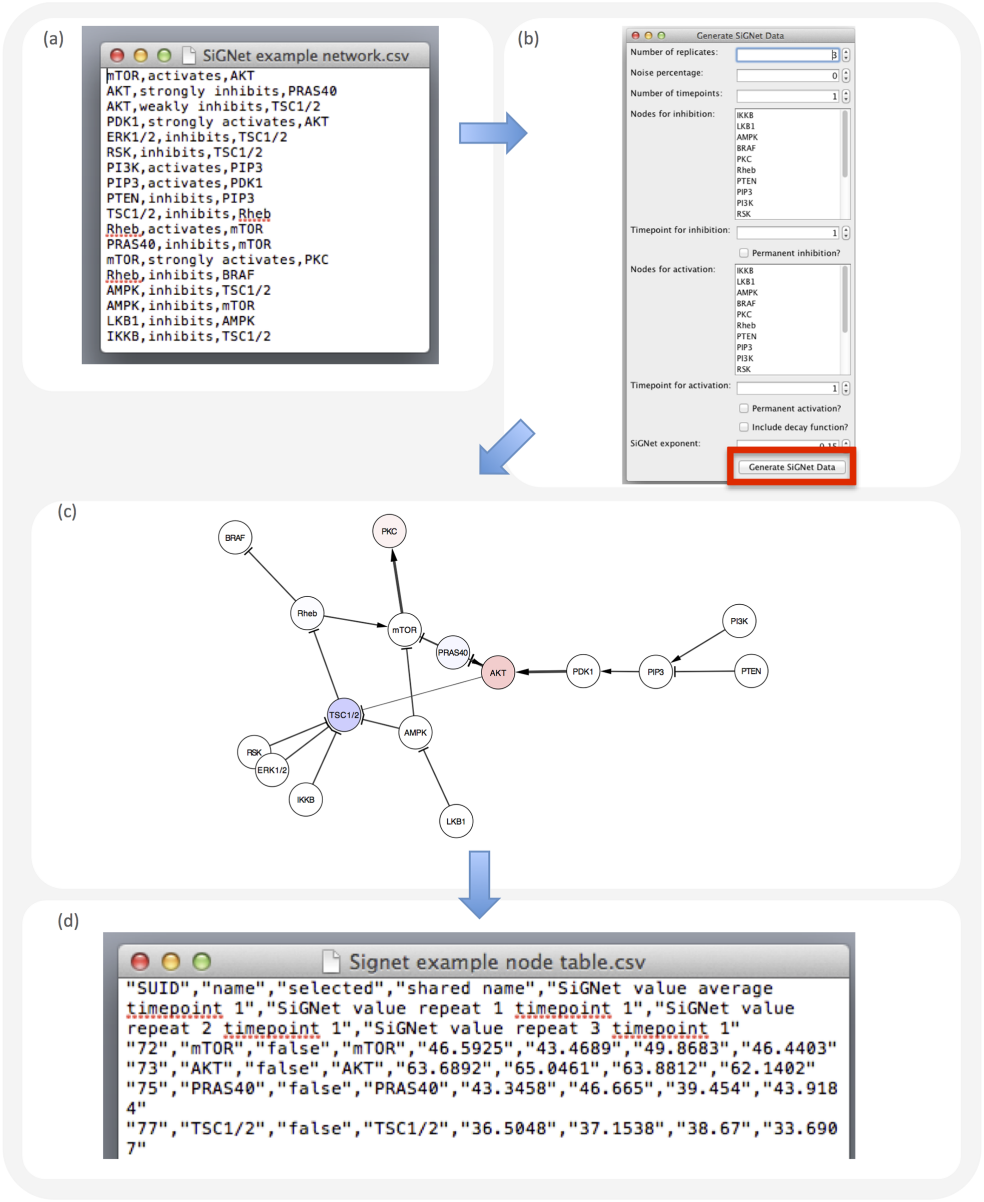
Using the SiGNet plugin to simulate experimental data for a biological network. **(A** to **D)** Screen snapshots showing how SiGNet is used. After a network is imported into or drawn out in Cytoscape (A), the user determines the number of ‘replicates’, ‘time points’, the amount of noise and whether nodes are inhibited or activated (B). SiGNet then generates simulated data for each replicate and time point. Here (C), nodes are colored according to their average value at time point 1. The data are exported and used for network inference (D). The accuracy of predicted edges can be benchmarked against the structure of the network used in A and scores of sensitivity, precision and recall can be calculated. A more detailed walkthrough example of how SiGNet can be used to aid benchmarking of inference techniques is available at signet.icr.ac.uk.

Here, we describe SiGNet (Signal Generator for Networks), a Cytoscape App [23] for generating in silico biological signaling data for the benchmarking of network inference approaches. A comparison of SiGNet with existing tools for generating transcriptional networks and simulated transcriptional datasets is shown in Table 1. Fig 1 presents an overview of how SiGNet is used. We have tested SiGNet using published experimental data, achieving an overall correlation between real and simulated data of 0.81, and we have used it to model key cancer-related pathways. The app is freely available for download in the Cytoscape App Store, and a typical use case is available at signet.icr.ac.uk.

**Table 1:** Comparing SiGNet to tools that simulate transcriptional network datasets.

Application name	Format	Type of network simulated	Network structure	Individual node dynamics	Can simulate knock-out/inhibition experiments?
**GeneNetWeaver**[17]	**Stand-alone Java application**	**Gene regulatory**	**Based on known gene regulatory networks of model organisms**	**Based on transcriptional regulatory dynamics incorporating protein and mRNA dynamics**	**✓**
**GRENDEL**[18]	**Stand-alone Java application**	**Gene regulatory**	**Randomly generated based on preferential attachment model**[18]	**Based on transcriptional regulatory dynamics of random gene in *S*. *cerevisiae*, incorporating protein and mRNA dynamics**	**✓**
**NetSim**[24]	**R package**	**Gene regulatory**	**Built from structural ‘modules’ enriched in *E*.*coli* and *S*.*cerevisiae* transcriptional networks**	**Modeled as an ordinary differential equation incorporating a sigmoidal activation function**	**✗**
**SynTReN**[25]	**Stand-alone Java application**	**Gene regulatory**	**Based on known gene regulatory networks of model organisms**	**Based on Michaelis-Menten and Hill enzyme kinetic equations**	**✗**
**SiGNet**	**Cytoscape app**	**Intracellular signaling network**	**Drawn out by user to their exact requirements or imported into Cytoscape from external source**	**Modeled as a sigmoidal stimulus-response curve, as observed in numerous cellular signaling systems**	**✓**

SiGNet is the only tool designed to simulate signalling data. In contrast to apps developed for simulating transcriptional network data, SiGNet allows the user to design the network for simulation, or import it into Cytoscape from an external source (Table 1).

## Results

### SiGNet incorporates user inputs to create bespoke simulated data

**Fig 2:**
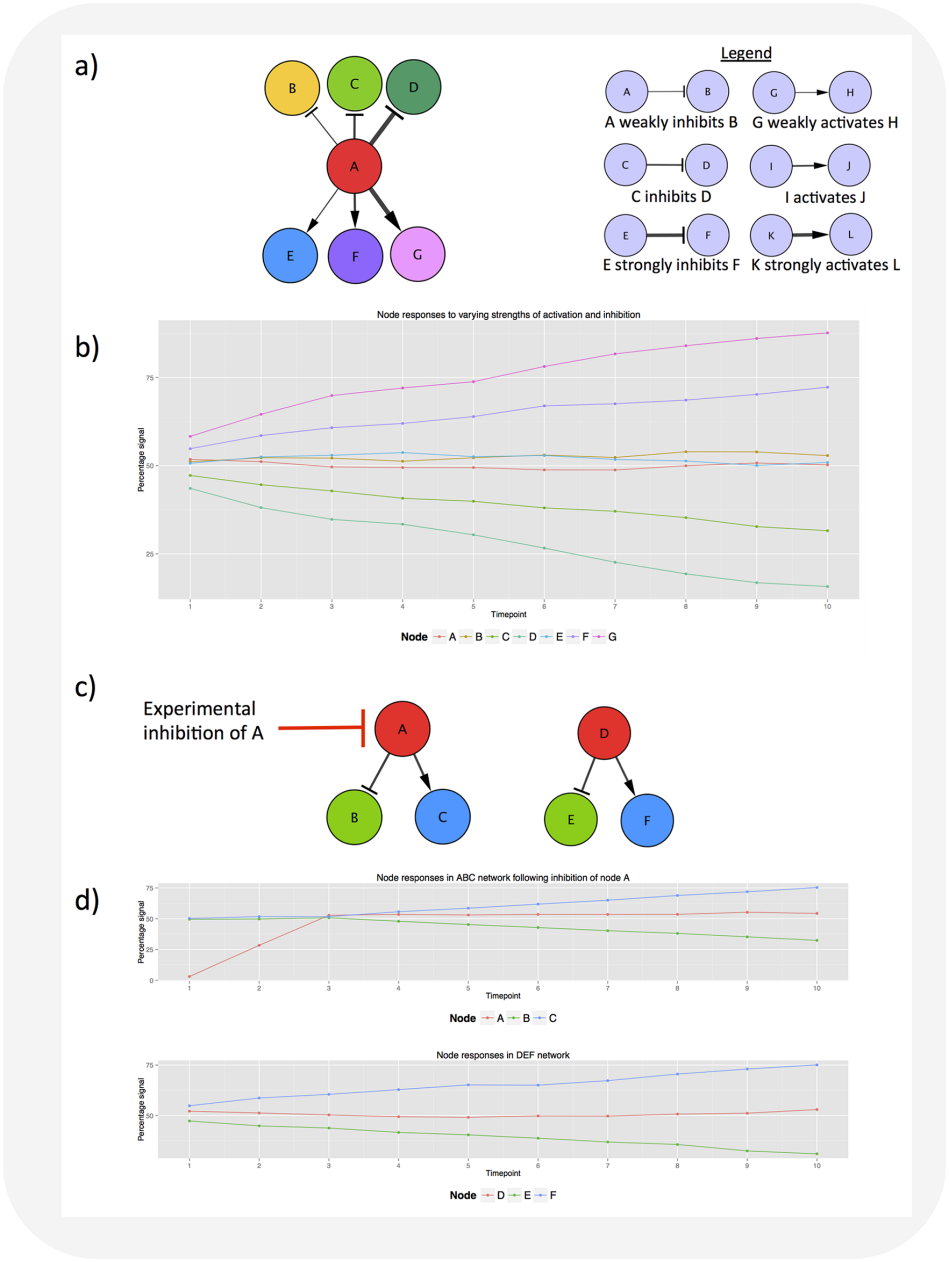
SiGNet simulates node responses to signaling interactions of different strengths and types. (**A**) Schematic showing the structure of a small network where node A weakly inhibits node B, inhibits node C, strongly inhibits node D, weakly activates node E, activates node F and strongly activates node G. (**B**) Graph showing the signaling output from each of the nodes shown in (A) over time, as predicted by SiGNet. Data are mean values calculated from ten ‘experimental replicates’ produced using SiGNet. (**C**) Schematic showing two networks, ABC and DEF, with identical network structures. The ABC network is perturbed by the experimental inhibition of A. (**D**) Graphs showing the signaling output of nodes A, B and C (upper) and nodes D, E and F (lower). The inhibition of node A is removed at time point 1.

In order to use SiGNet, a user must import or define a network structure, including variables dictating the nature and strength of interactions between nodes (Fig 2A). The number of ‘experimental repeats’ required and the level of noise (stochasticity) in the system must also be specified. The nature and strength of an interaction is supplied in controlled vocabulary: ‘activates’, ‘weakly activates’, ‘strongly activates’, ‘inhibits’, ‘weakly inhibits’, ‘strongly inhibits’, or ‘binds’ (for interactions where nodes do not affect each other’s activity). Example input files are included in the S1 File. If an interaction is described using a term not in this controlled vocabulary, SiGNet will replace the term with ‘activates’ and this will be reported to the user. Using this information, SiGNet generates graded responses to the specified interactions (Fig 2B). Users are also able to identify which, if any, network nodes they wish to be subject to ‘external’ inhibition or activation (e.g. to mimic targeting by a drug) (Fig 2C) and then assess the effect of this on signaling output (Fig 2D).

SiGNet assigns each node in the network a value between 0 and 100%, reflecting the percentage activity of the protein: this is a biologically appropriate assumption as data from laboratory experiments are normalized to baseline levels [26]. Unless a node has been selected as inhibited or activated, nodes will initially be assigned an initial baseline level of activity of approximately 50%, reflecting the homeostatic, steady state of a cell. Users also have the option to use their own data to inform the baseline protein levels—a tutorial for this is available at http://signet.icr.ac.uk. Different forms of the same protein (splice variants, phosphorylated proteins etc.) should be represented as separate nodes within the network.

### SiGNet signals simulate real biological behaviour

Many of the reactions underlying cellular signaling networks are non-linear. For example, enzymatic reactions are frequently modeled using the non-linear Michaelis-Menten equation [27], whilst the reversible covalent modification of proteins (e.g. phosphorylation) is often modeled using the non-linear Goldbeter-Koshland kinetic model [28]. Graded and reversible signaling responses can often be represented by sigmoidal stimulus-response curves. For example, sigmoidal stimulus-response curves can describe multistep signaling, zero-order ultra sensitivity (covalent modification) and positive feedback [29]. For these reasons, we chose a sigmoidal signal-response curve as the general case for SiGNet:

$$y=\left(\frac{100}{1+e^{-0.15x}}\right)-50$$

where x = net input into the node (‘signal’) and y = change in node activity (‘response’).

A similar formula is applied in SiGNet to model the loss of protein activity (decay) over time:

$$y=\left(\frac{100}{1+e^{0.15x}}\right)-50$$

where x = a randomly generated number between 0 and 5, and y = change in protein activity (‘response’). The optional decay function applies to all nodes in the network and is an implicit decay that is not regulated by other proteins present. This represents the natural turnover and degradation of proteins within the cell and enables the system to eventually return to homeostasis after stimulation. x is chosen randomly to ensure that different proteins will decay at different rates—a realistic assumption as protein degradation is a stochastic process. The decay function can only be used for simulations with time series of two or more time points, and is applied only for the second half of the overall time series. The value of the exponent in both the signal-response and decay functions is set at 0.15 by default—if desired, the user may change this value in the SiGNet interface.

Due to the sigmoidal signal-response curve used in the SiGNet algorithm, it is assumed that all node responses are continuous and do not form a one-way switch or ‘point of no return’ such as a cell cycle checkpoint. For this reason, it is also assumed that the nodes in the network are not spatially restrained and are evenly distributed throughout the cell, at saturation.

### SiGNet simulates stochasticity in the signaling network

Noise due to stochastic fluctuations in concentration has been observed in both transcriptional [30] and signaling networks [31]. In signaling networks, this noise may be due to a number of processes, including protein promiscuity and transient nonspecific protein-protein interactions. SiGNet allows stochasticity to be incorporated into the data simulation: the term ‘stochasticity’ in this context refers to the level of noise in the relationship between node input and the change in node activation, and can be specified by the user (default noise level is 0%). This enables benchmarking at multiple noise levels to test the robustness of the performance of the inferred network. S1 Fig demonstrates the relationship between input (i.e. activating or inhibitory signal going into a node) and output (the change in activity of the node).

### SiGNet simulations can replicate real protein dynamics

**Fig 3:**
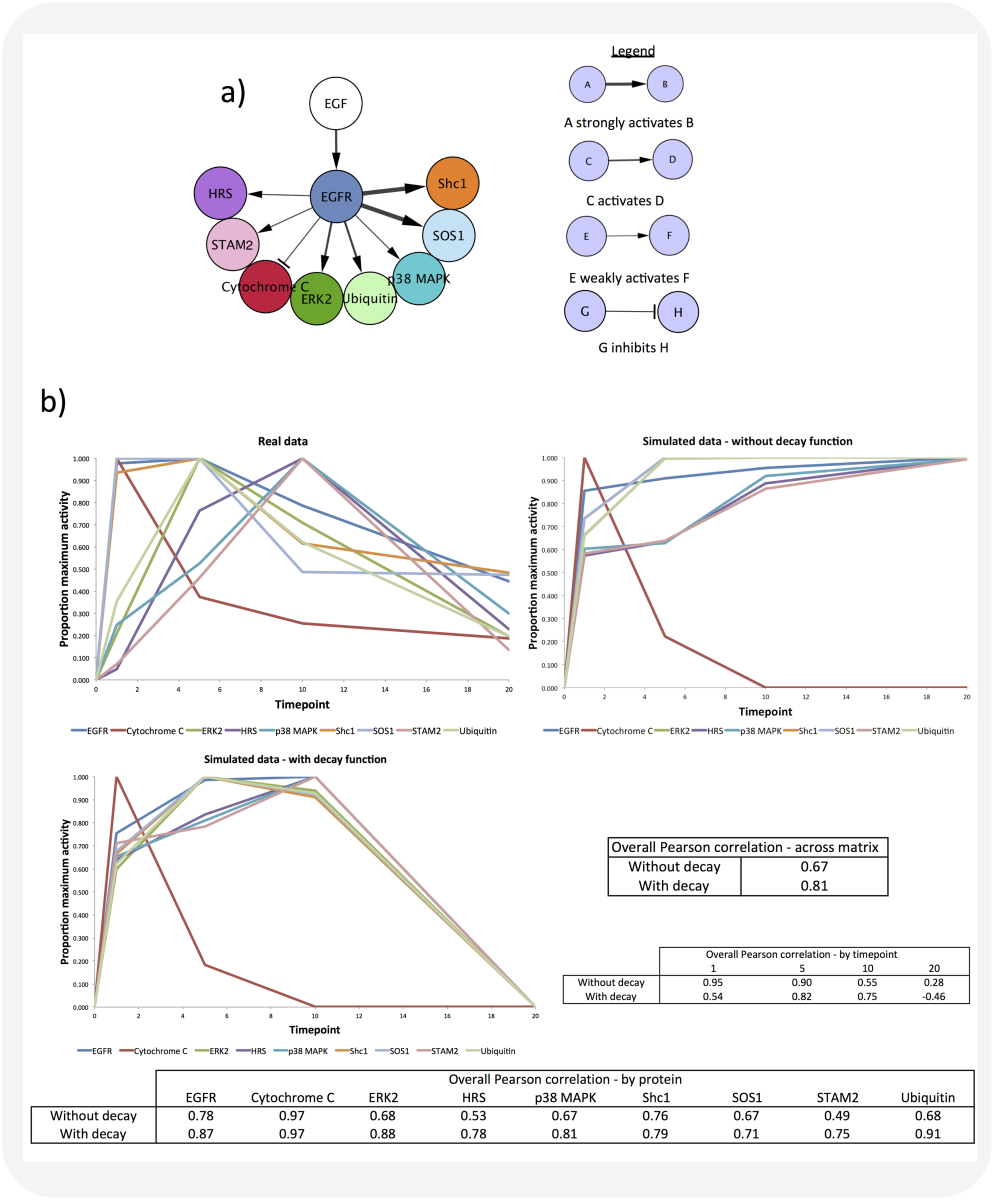
Simulating the effect of experimental perturbation on a real biological signaling network in SiGNet. SiGNet was used to simulate the effect of EGF treatment on EGFR and its downstream proteins, and the simulated data tested against published experimental data [32]. (**A**) Schematic showing the network structure, which was based on interactions reported by Blagoev et al. The network was drawn in Cytoscape and used as an input for SiGNet. (**B**) Data simulated for the network in (A) using SiGNet, with and without the optional decay function. Data shown are mean values calculated from ten ‘experimental replicates’. Pearson correlations between simulated and real data are shown. Additional simulations of this network incorporating simple feedback mechanisms are illustrated in S2, S3 and S4 Figs.

A widely-cited example of a quantitative phosphoproteomics study [32] shows the effect of EGF treatment on the dynamic behavior of small protein networks in HeLa cells. We have used SiGNet to simulate these data and achieved a Pearson correlation with the real data of up to 0.97 (Fig 3). The network depicted in Fig 3A shows the interactions between EGFR and a number of downstream proteins, as described in [32]. The strengths of the interactions in the simulation of this network were based on experimental data of the protein dynamics, shown in Fig 3. For example, the EGFR-STAM2 interaction was assigned a ‘weakly activates’ strength as STAM2 activity increases slowly following EGF treatment, compared to Shc1, which increases its activity rapidly following EGF treatment (the EGFR-Shc1 interaction is assigned a strength of “strongly activates”). Data from this network were simulated in SiGNet and benchmarking (not shown) indicated that the best fit between simulated and real data was achieved when one SiGNet time-step equated to 0.5 minutes. When our simulated data was compared to the real data, we found that incorporating the decay function into the SiGNet model increased the correlation between the simulated and real data for all proteins, but not at all time points (Fig 3B). Overall, the SiGNet data generated with the decay function correlated with the real data with a Pearson correlation of 0.81, and that generated without the decay function had a Pearson correlation of 0.67. Further simulations of this network incorporating simple feedback mechanisms are illustrated in S2, S3 and S4 Figs: these simulations produce results that are consistent with (although not identical to) experimental data. Reassuringly, the strength of the inhibitory feedback loop is reflected in the response of Cytochrome C. In S3 Fig, inhibition of EGFR by the feedback loop partially relieves the inhibition of EGFR on Cytochrome C, resulting in Cytochrome C’s activity falling in response to EGFR inhibition, followed by a gradual increase in activity over time steps 5–20 as inhibition of the inhibition occurs. In S4 Fig, where the feedback loop applies a strong inhibitory response to EGFR, Cytochrome C maintains high activity throughout the simulation, corresponding to almost complete relief of the inhibitory activity of EGFR on Cytochrome C. However, addition of the feedback loops may increase the accuracy of simulations of one protein, whilst decreasing the accuracy of simulation of other parts of the network. By adding feedback loops or additional control motifs to the network to be simulated, the user can investigate the effect of these elements on the activity of the network as a whole. If addition of a feedback loop decreases the accuracy of a simulation, this suggests that a feedback loop as modeled in SiGNet will not be responsible for the experimental data for the observed network. This demonstrates how SiGNet can be used to identify gaps in published signaling networks and highlight potential interactions for further experimental characterization.

We have also used SiGNet to reconstruct key signaling networks based on KEGG (Kyoto Encyclopedia of Genes and Genomes) pathways [33], namely PI3K, mTOR and Ras. The input files and simulated data for these networks can be found at signet.icr.ac.uk. For each of the three pathways modeled there are Readme files detailing how the networks were constructed, with hyperlinks to the relevant KEGG pathway, information on the files supplied, and details of the SiGNet parameters used to simulate the data supplied. All interactions are described using either ‘inhibits’ or ‘activates’ (no ‘strongly’/‘weakly’) as the KEGG pathway figures used to construct the networks do not quantify the strength of interactions.

As discussed regarding the strengths of interactions used to generate the simulated data in Fig 3, small amounts of experimental data, when available, can be used to help design the initial network used as input for SiGNet. We would encourage the user to adjust the various parameters in SiGNet according to any other prior knowledge they have of the system, for example by loading a baseline activation profile for the proteins in the network. We would also recommend generating multiple simulations, for example simulating multiple data sets with various noise percentages, to enable more robust assessment of the quality of inference techniques. We believe that SiGNet enables the rapid generation of large amounts of bespoke simulated data, and as such enables detailed and reliable assessment of network inference strategies. For a detailed discussion of the data sources available for assessing the accuracy of SiGNet simulations, including the simulations of data from [34], see S1 File.

## Discussion

SiGNet enables researchers to create realistic, bespoke benchmarking datasets for the evaluation of signaling network inference, and an example of how SiGNet can be used in this way is presented on the SiGNet website. The SiGNet algorithm is based on our understanding of protein behavior, in contrast to similar tools developed for transcriptional networks which are designed to mimic the dynamics of transcription factors and their target genes. SiGNet users can specify the type and strength of interactions within a signaling network of their own design. The app also includes options for inhibiting or activating nodes, mimicking experimental peturbation. When we used SiGNet to reproduce data from real experiments its simulations were highly accurate, with correlations between real and simulated data of up to 0.97, although identifying datasets suitable for this validation represented a major challenge.

It is difficult to assess the accuracy of a data simulator such as SiGNet due to a Catch-22 situation: if experimental datasets existed that were suitable for the task then there would be no need to create the simulator in the first place. However, we have validated our simulations against the best available datasets and found our simulations to be accurate. There are three main issues in identifying suitable datasets. Firstly, the lack of quantitative proteomics measurements reflects biologists’ widespread reliance on non-quantitative techniques. Secondly, the lack of data for a complete protein network is, at least in part, due to the relative scarcity of large-scale, proteomics studies. The larger the number of proteins studied, the more likely there is to be a complete protein-protein interaction network present within the data. Thirdly, a lack of dynamic, time-source data reflects the challenge of obtaining large numbers of experimental data points. Until such datasets become available, SiGNet provides a valuable resource which demonstrates strong concordance with available published data. We have used SiGNet to model important cancer-related pathways and provide the simulated data as a public resource. In addition to its use in benchmarking inference strategies, SiGNet could be used to develop initial models and hypotheses regarding the behavior of signaling networks following genetic or pharmacological perturbation.

## Materials and methods

SiGNet is a Java-based Cytoscape [23] plugin. It is compatible with Cytoscape Version 3.2.0+ and is available for download in the Cytoscape App Store (http://apps.cytoscape.org). Detailed documentation including example data and a walkthrough is available at signet.icr.ac.uk.

Code is available at https://github.com/eac54/SiGNet and supporting data and documentation is available at https://figshare.com/articles/SiGNet_a_signaling_network_data_simulator_to_enable_signaling_network_inference/4578808. SiGNet is licensed under the Creative Commons Attribution-ShareAlike 4.0 International License. To view a copy of this license, visit http://creativecommons.org/licenses/by-sa/4.0/.

## Supporting information

S1 Fig SiGNet simulations predict how noise affects signaling inputs and outputs. Data points are mean values from ten ‘experimental replicates’ produced using SiGNet, incorporating a user-specified amount of noise. Negative inputs correspond to node inhibition; positive inputs correspond to node activation. Inputs range from ‘weak’ (0.5) to ‘strong’ (1.5). For each node, net input is calculated as the total score of activating interactions minus the total score of inhibitory interactions.
(TIFF)

S2 Fig Results of simulations of network shown in Fig 3, with an additional weak feedback loop modeled. SiGNet was used to simulate the effect of EGF treatment on EGFR and its downstream proteins, and the simulated data tested against published experimental data [32], with addition of simple, generic feedback loop nodes. These simulations with a simple feedback mechanism generally show poorer Pearson correlations between the simulated and real data than the modeling done in Fig 3 of the main manuscript. This demonstrates that adding a simple feedback loop to the network does not improve the accuracy of the data simulation and hence it is unlikely that such simple feedback loops are responsible for the experimental observations. More complex, multi-component feedback loops could be constructed, for example based upon additional experimental data, and simulated to identify and prioritise possible ‘missing’ interactions in the network. (**A**) Schematic showing the network structure, which was based on interactions reported by Blagoev et al, with an additional feedback loop added. Here this corresponds to a node activated by ERK that weakly inhibits EGFR. The network was drawn in Cytoscape and used as an input for SiGNet. (**B**) Data simulated for the network in (A) using SiGNet. Data shown are mean values calculated from ten ‘experimental replicates’ and without the use of the optional decay function. Pearson correlations between simulated and real data are shown. (**C**) Data simulated as per (**B**), applying the optional decay function. Pearson correlations between simulated and real data are shown.
(TIFF)

S3 Fig Results of simulations of network shown in Fig 3, with an additional feedback loop modeled. SiGNet was used to simulate the effect of EGF treatment on EGFR and its downstream proteins, and the simulated data tested against published experimental data [32], with addition of simple, generic feedback loop nodes. These simulations with a simple feedback mechanism generally show poorer Pearson correlations between the simulated and real data than the modeling done in Fig 3 of the main manuscript. This demonstrates that adding a simple feedback loop to the network does not improve the accuracy of the data simulation and hence it is unlikely that such simple feedback loops are responsible for the experimental observations. More complex, multi-component feedback loops could be constructed, for example based upon additional experimental data, and simulated to identify and prioritise possible ‘missing’ interactions in the network. (**A**) Schematic showing the network structure, which was based on interactions reported by Blagoev et al, with an additional feedback loop added. Herethis corresponds to a node activated by ERK that inhibits EGFR at standard strength. The network was drawn in Cytoscape and used as an input for SiGNet. (**B**) Data simulated for the network in (A) using SiGNet. Data shown are mean values calculated from ten ‘experimental replicates’ and without the use of the optional decay function. Pearson correlations between simulated and real data are shown. (**C**) Data simulated as per (**B**), applying the optional decay function. Pearson correlations between simulated and real data are shown.
(TIFF)

S4 Fig Results of simulations of network shown in Fig 3, with an additional strong feedback loops modeled. SiGNet was used to simulate the effect of EGF treatment on EGFR and its downstream proteins, and the simulated data tested against published experimental data [32], with addition of simple, generic feedback loop nodes. These simulations with a simple feedback mechanism generally show poorer Pearson correlations between the simulated and real data than the modeling done in Fig 3 of the main manuscript. This demonstrates that adding a simple feedback loop to the network does not improve the accuracy of the data simulation and hence it is unlikely that such simple feedback loops are responsible for the experimental observations. More complex, multi-component feedback loops could be constructed, for example based upon additional experimental data, and simulated to identify and prioritise possible ‘missing’ interactions in the network. (**A**) Schematic showing the network structure, which was based on interactions reported by Blagoev et al, with an additional feedback loop added. Here this corresponds to a node activated by ERK that strongly inhibits EGFR. The network was drawn in Cytoscape and used as an input for SiGNet. (**B**) Data simulated for the network in (A) using SiGNet. Data shown are mean values calculated from ten ‘experimental replicates’ and without the use of the optional decay function. Pearson correlations between simulated and real data are shown. (**C**) Data simulated as per (**B**), applying the optional decay function. Pearson correlations between simulated and real data are shown.
(TIFF)

S5 Fig Using SiGNet to reproduce experimental protein dynamics - simulating protein phosphorylation within network motifs. SiGNet was used to simulate the effect of EGF treatment on protein phosphorylation within HeLa cells, and the simulated data tested against published experimental data [33]. (**A**) Normalised proteomics data for a number of network motifs (B). Data is reproduced, with permission from Elsevier Ltd, from a 2006 paper published by Olsen et al [33]. (**B**) Schematic representation of the network motifs [33]. (**C**) Simulated data generated by SiGNet for the network motifs shown in (B). Data shown are mean values of ten ‘experimental replicates’.
(TIFF)

S1 File Discussion of the difficulty of obtaining suitable data for assessing SiGNet’s accuracy. (DOCX)
