# Supplementary figures and images for: SiGNet: A signaling network data simulator to enable signaling network inference
Source: PLoS One. 2017 May 17;12(5):e0177701. doi: 10.1371/journal.pone.0177701 (PMC5435248; doi:10.1371/journal.pone.0177701)

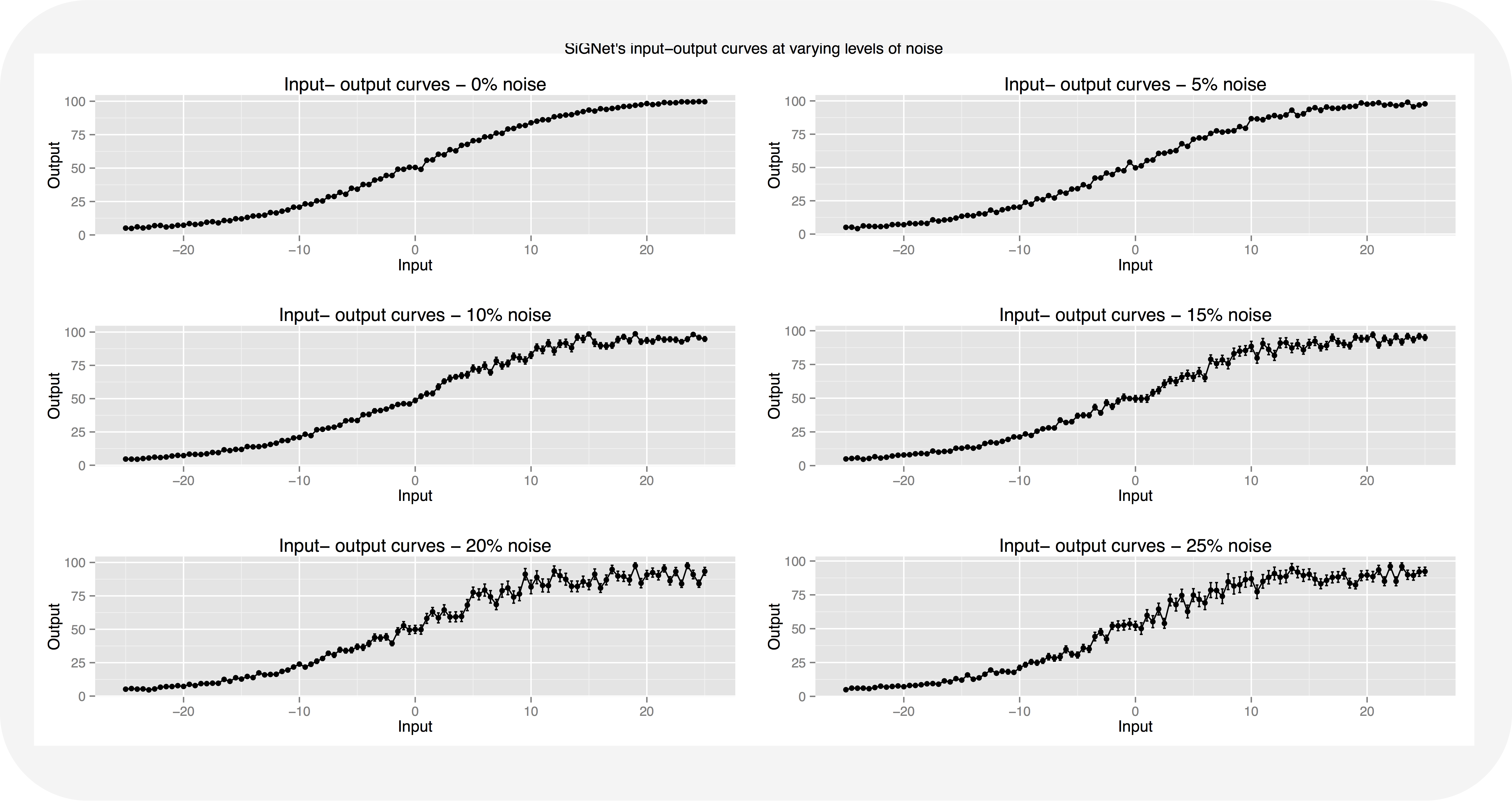

Supplement: S1 Fig — Data points are mean values from ten ‘experimental replicates’ produced using SiGNet, incorporating a user-specified amount of noise. Negative inputs correspond to node inhibition; positive inputs correspond to node activation. Inputs range from ‘weak’ (0.5) to ‘strong’ (1.5). For each node, net input is calculated as the total score of activating interactions minus the total score of inhibitory interactions. (TIFF) [file pone.0177701.s001.tiff]

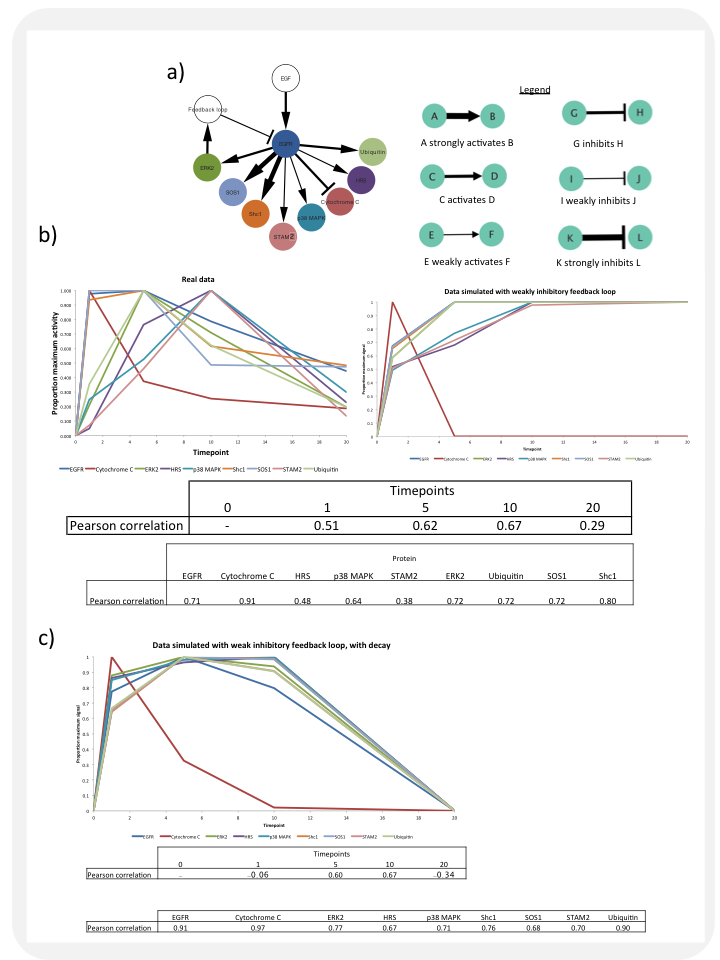

Supplement: S2 Fig — SiGNet was used to simulate the effect of EGF treatment on EGFR and its downstream proteins, and the simulated data tested against published experimental data [32], with addition of simple, generic feedback loop nodes. These simulations with a simple feedback mechanism generally show poorer Pearson correlations between the simulated and real data than the modeling done in Fig 3 of the main manuscript. This demonstrates that adding a simple feedback loop to the network does not improve the accuracy of the data simulation and hence it is unlikely that such simple feedback loops are responsible for the experimental observations. More complex, multi-component feedback loops could be constructed, for example based upon additional experimental data, and simulated to identify and prioritise possible ‘missing’ interactions in the network. (A) Schematic showing the network structure, which was based on interactions reported by Blagoev et al, with an additional feedback loop added. Here this corresponds to a node activated by ERK that weakly inhibits EGFR. The network was drawn in Cytoscape and used as an input for SiGNet. (B) Data simulated for the network in (A) using SiGNet. Data shown are mean values calculated from ten ‘experimental replicates’ and without the use of the optional decay function. Pearson correlations between simulated and real data are shown. (C) Data simulated as per (B), applying the optional decay function. Pearson correlations between simulated and real data are shown. (TIFF) [file pone.0177701.s002.tiff]

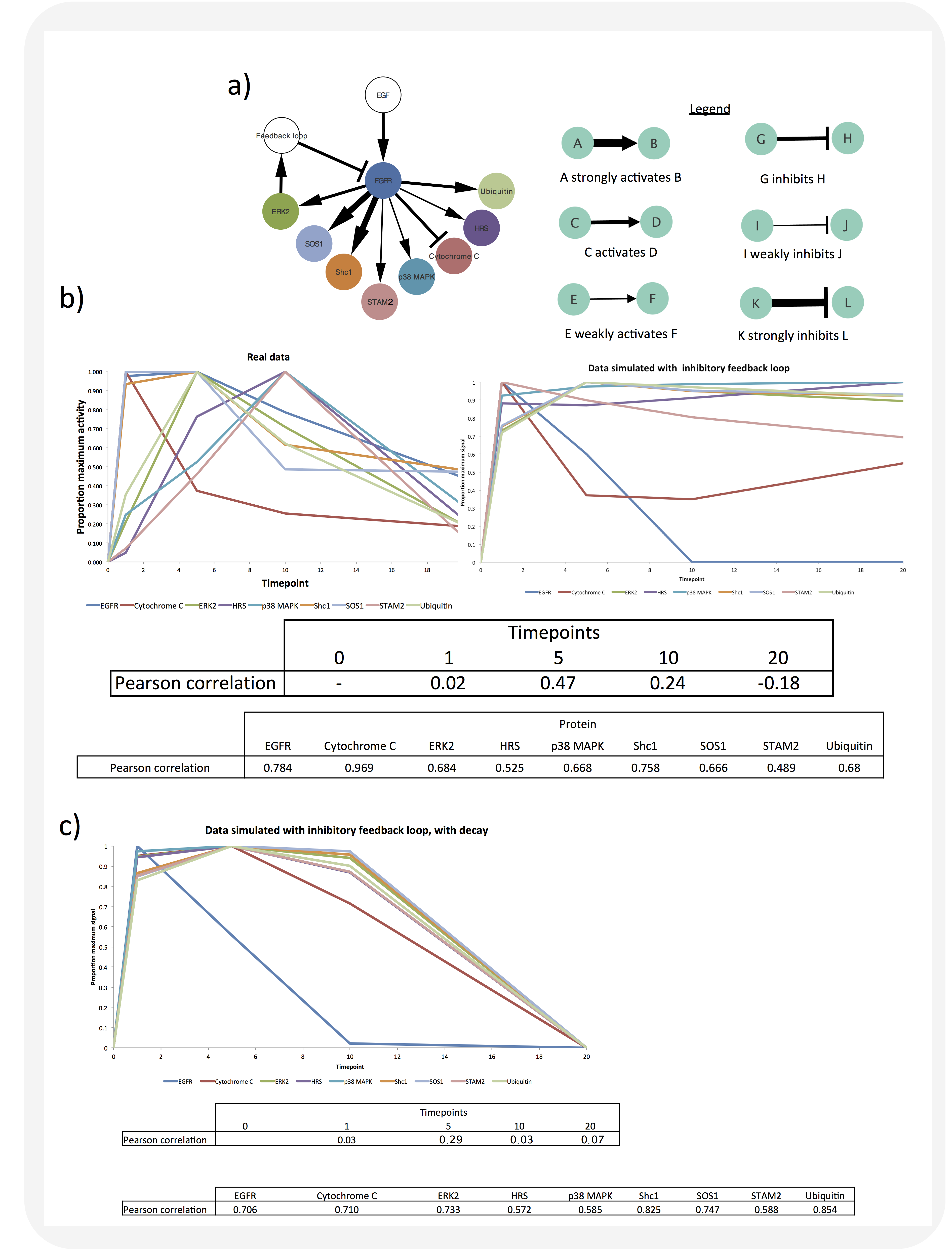

Supplement: S3 Fig — SiGNet was used to simulate the effect of EGF treatment on EGFR and its downstream proteins, and the simulated data tested against published experimental data [32], with addition of simple, generic feedback loop nodes. These simulations with a simple feedback mechanism generally show poorer Pearson correlations between the simulated and real data than the modeling done in Fig 3 of the main manuscript. This demonstrates that adding a simple feedback loop to the network does not improve the accuracy of the data simulation and hence it is unlikely that such simple feedback loops are responsible for the experimental observations. More complex, multi-component feedback loops could be constructed, for example based upon additional experimental data, and simulated to identify and prioritise possible ‘missing’ interactions in the network. (A) Schematic showing the network structure, which was based on interactions reported by Blagoev et al, with an additional feedback loop added. Herethis corresponds to a node activated by ERK that inhibits EGFR at standard strength. The network was drawn in Cytoscape and used as an input for SiGNet. (B) Data simulated for the network in (A) using SiGNet. Data shown are mean values calculated from ten ‘experimental replicates’ and without the use of the optional decay function. Pearson correlations between simulated and real data are shown. (C) Data simulated as per (B), applying the optional decay function. Pearson correlations between simulated and real data are shown. (TIFF) [file pone.0177701.s003.tiff]

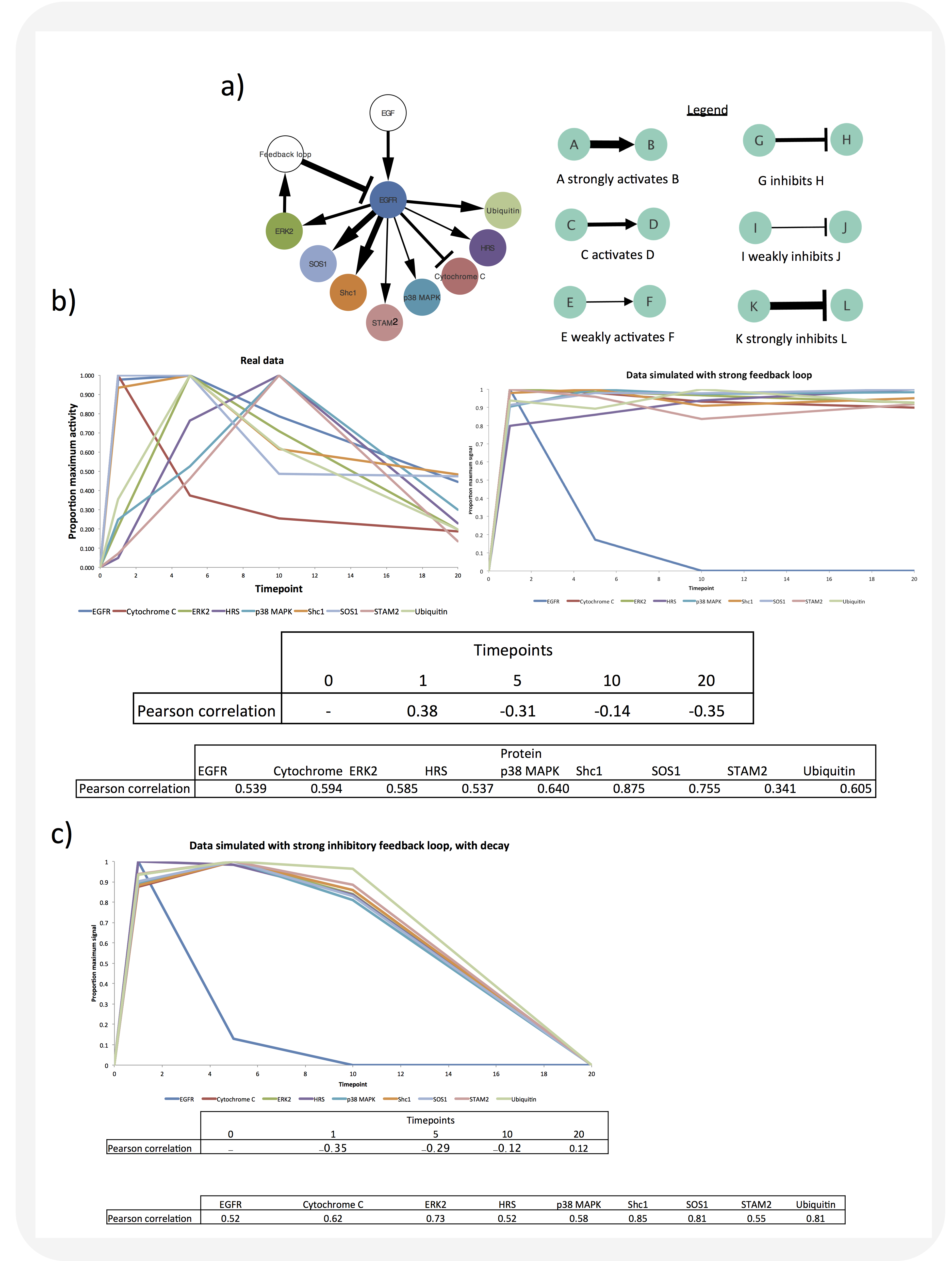

Supplement: S4 Fig — SiGNet was used to simulate the effect of EGF treatment on EGFR and its downstream proteins, and the simulated data tested against published experimental data [32], with addition of simple, generic feedback loop nodes. These simulations with a simple feedback mechanism generally show poorer Pearson correlations between the simulated and real data than the modeling done in Fig 3 of the main manuscript. This demonstrates that adding a simple feedback loop to the network does not improve the accuracy of the data simulation and hence it is unlikely that such simple feedback loops are responsible for the experimental observations. More complex, multi-component feedback loops could be constructed, for example based upon additional experimental data, and simulated to identify and prioritise possible ‘missing’ interactions in the network. (A) Schematic showing the network structure, which was based on interactions reported by Blagoev et al, with an additional feedback loop added. Here this corresponds to a node activated by ERK that strongly inhibits EGFR. The network was drawn in Cytoscape and used as an input for SiGNet. (B) Data simulated for the network in (A) using SiGNet. Data shown are mean values calculated from ten ‘experimental replicates’ and without the use of the optional decay function. Pearson correlations between simulated and real data are shown. (C) Data simulated as per (B), applying the optional decay function. Pearson correlations between simulated and real data are shown. (TIFF) [file pone.0177701.s004.tiff]

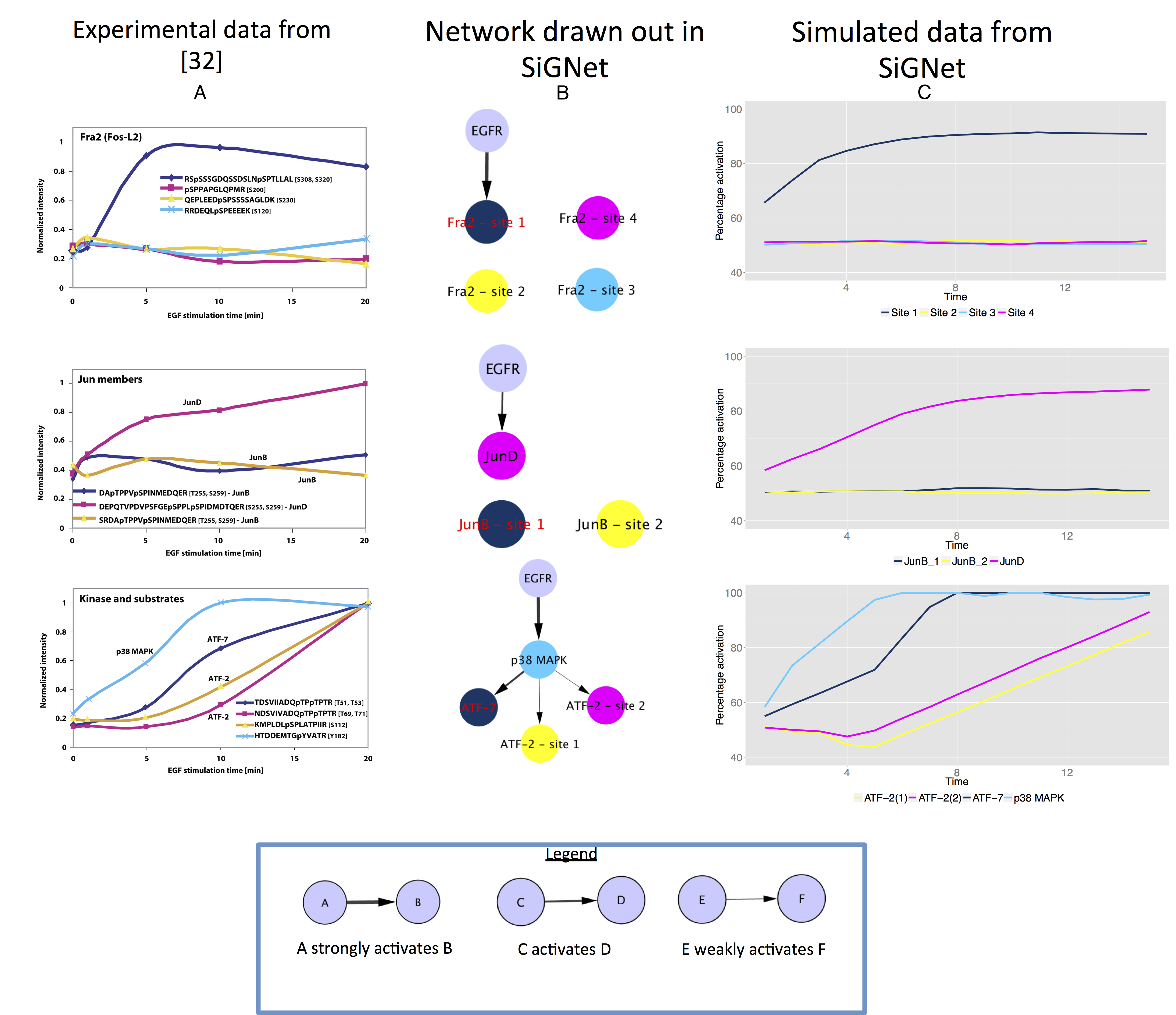

Supplement: S5 Fig — SiGNet was used to simulate the effect of EGF treatment on protein phosphorylation within HeLa cells, and the simulated data tested against published experimental data [33]. (A) Normalised proteomics data for a number of network motifs (B). Data is reproduced, with permission from Elsevier Ltd, from a 2006 paper published by Olsen et al [33]. (B) Schematic representation of the network motifs [33]. (C) Simulated data generated by SiGNet for the network motifs shown in (B). Data shown are mean values of ten ‘experimental replicates’. (TIFF) [file pone.0177701.s005.tiff]
