# Supplementary material for: SiGNet: A signaling network data simulator to enable signaling network inference
Source: PLoS One. 2017 May 17;12(5):e0177701. doi: 10.1371/journal.pone.0177701 (PMC5435248; doi:10.1371/journal.pone.0177701)
Supplement: S1 File — (DOCX) [file pone.0177701.s006.docx]

**S1 File: Supplementary discussion of data sources for benchmarking**

To test the accuracy of SiGNet data simulations, it is necessary to have experimental data of the same format as that produced by the SiGNet algorithm, i.e. quantitative measurements of proteins in a network of known structure, measured over multiple time points. However, if such data sources were widely available there would be no need to build a data simulator to produce such data for benchmarking of inference strategies – a Catch-22 situation.

Here we discuss a number of data sources we have considered for testing the accuracy of SiGNet data simulations, and their limitations. The papers discussed here have been chosen as representative examples of common issues.

**Lack of quantitative data**

Traditionally, perturbations in the levels of signaling proteins resulting from the application of targeted drugs or siRNA are demonstrated by Western blotting. However, this technique provides only semi-quantitative data unless appropriate calibration procedures have been used and described [1]. Although journals are placing increasing emphasis on the quantification of Western blots[2], it is still rare to find quantitative protein data for a complete signaling network of known structure. In addition to this, Western blotting only indicates the abundance of the protein of interest, which does not necessarily correlate with protein activity. For this reason, such studies are unsuitable for assessing the accuracy of the SiGNet algorithm.

**Lack of complete networks**

The SiGNet algorithm produces a value for the abundance of a particular protein based on the structure and nature of its surrounding network, as specified in Cytoscape. This value is therefore highly dependent on the behavior of the other proteins in the network. This means that to assess the performance of SiGNet simulations, proteomics data is required for a complete network of known interactions without orphan (unconnected) nodes. This renders studies, such as that by Niepel and coworkers[3], with data freely available to download from the LINCS Database[4], unsuitable for our purposes. Their study uses a large panel of 39 cell lines, treated with 15 growth factors at three time points and two doses, yet only measures the phosphorylation level of two proteins – pERK and pAKT. Two nodes do not form a network so this paper, and ones like it, are unsuitable for assessing the accuracy of the SiGNet algorithm.

Other datasets that may otherwise appear ideal for benchmarking of the SiGNet algorithm, may be missing vital data. For example, the ‘Phosphorylation state and protein levels measured in BRAF(V600E/D) melanoma cell lines monitored by Reverse Phase Protein Arrays’ dataset[5] (entry 20218 in the LINCS database[4]) provides measurements of a number of phosphorylated proteins such as MEK and AKT following treatment with one of several BRAF inhibitors at multiple time points. However, activation/phosphorylation of the BRAF protein was not measured in the project, and so experimental data for the BRAF node is not available. SiGNet can only simulate inhibition of nodes present in the network entered into Cytoscape, and whilst SiGNet would be able to generate data for a BRAF node artificially added to the network, it would not be possible to assess the accuracy of this simulation. As the activity of the BRAF protein is, by definition, crucial to the activity of other proteins in the network, this experimental dataset would not provide a meaningful dataset for assessing the accuracy of SiGNet algorithm.

Another side to this issue is the lack of specificity in known interactomes. Many mass spectrometry studies publish data for the phosphorylation of peptides at specific sites[6]. However, little data is available for the phosphorylation that occurs as a result of site-specific protein-protein interactions. Using information found within the Phosphosite Database of kinase-substrate interactions[7] it is apparent that in the Wolf-Yadlin et al study only 21.7% of the phosphorylation sites measured using MRM-QTRAP have known kinases, and only 30.3% of the phosphopeptides measured using IDA-QSTAR have known kinases. In addition to this, even when a kinase-substrate relationship is known, it is not stated whether the interaction is activatory or inhibitory. This therefore presents another major challenge in identifying suitable datasets for assessing the accuracy of SiGNet algorithm simulations.

**Lack of Multiple Time Points**

The larger the number of proteins measured in a study, the more likely it is that there will be complete, continuous network motifs present in the data. However, for practical reasons proteomics study usually focus on maximising either the breadth of the study (i.e. measuring large numbers of proteins) or depth (i.e. multiple time points, perturbation by multiple drugs/ligands etc). A recent example of a large-scale quantitative proteomic analysis [8], based on 28 prostate tumour and eight non-malignant samples, identified and quantified over nine thousand proteins. This study is designed to produce a single timepoint, ‘snapshot’ of the proteome, rather than to measure the dynamics of a protein system over time.

Another dataset representing the activity of a number of proteins in a network at a single time point is the basal profile of receptor tyrosine kinase signaling network measured by ELISA dataset[3, 9] (entry 20137 in the LINCS database[4]). Single time point datasets are widely available, but as these data do not describe the behaviour of a system over time, they are unsuitable for assessing the accuracy of the SiGNet algorithm.

**Example: Global, in vivo, and site-specific phosphorylation dynamics in signaling networks**

The data presented by Olsen et al [10] in their 2006 paper appears to fulfil the requirements of a test dataset for SiGNet, particularly the data presented in the paper’s figure 5. SiGNet is able to simulate data that visually resembles the plots provided by Olsen et al in their original paper (S5 Fig). Using the Supplementary Data provided by Olsen et al and their online database Phosida[11, 12], we were able to retrieve an incomplete set of quantitative data for the markers shown in S5 Fig. However, these did not correspond to the plots shown by Olsen et al in terms of the shape of the response and the number of data points plotted. As such, we determined this dataset was unsuitable for use in quantitatively assessing the accuracy of SiGNet simulations, although the response profiles generated by the simulation resemble those published in graphical form by Olsen et al.

**Concluding remarks**

Assessing the accuracy of a data simulator such as SiGNet is a Catch-22 situation: if experimental datasets existed that were suitable for testing the algorithm’s accuracy, there would be no need to create the simulator in the first place. There are three main issues in identifying suitable datasets for this task. The first, the lack of quantitative proteomics measurements, reflects biologists’ widespread reliance on non-quantitative techniques such as Western blots. The second, the lack of complete networks of proteins, is at least in part due to the relative scarcity of large-scale, global proteomics studies. The larger the number of proteins studied, the more likely there is to be a complete protein-protein interaction network present within the proteins. The third issue, that of a lack of dynamic, time-source data, also reflects the challenge of obtaining larges amounts of experimental data points. Researchers are frequently required to decide between studying multiple proteins at a single time point, or fewer proteins at multiple time points. We have been unable to find experimental data that has both large numbers of data points and multiple time points, and therefore have been unable to identify data that meets all of the requirements for testing SiGNet’s simulation accuracy. However, as the SiGNet algorithm has been designed to closely mimic known behaviours of proteins, we believe the SiGNet Cytoscape app to be a valuable tool for the signalling and network biology research communities.

**References**:

[1] R.M. Murphy, G.D. Lamb, Important considerations for protein analyses using antibody based techniques: down-sizing Western blotting up-sizes outcomes, J Physiol 591(Pt 23) (2013) 5823-31.

[2] S.C. Taylor, T. Berkelman, G. Yadav, M. Hammond, A defined methodology for reliable quantification of Western blot data, Mol Biotechnol 55(3) (2013) 217-26.

[3] M. Niepel, M. Hafner, E.A. Pace, M. Chung, D.H. Chai, L. Zhou, J.L. Muhlich, B. Schoeberl, P.K. Sorger, Analysis of growth factor signaling in genetically diverse breast cancer lines, BMC Biol 12 (2014) 20.

[4] NIH Library of Integrated Network-Based Cellular Signatures (LINCS) Program – <http://www.lincsproject.org>.).

[5] M. Fallahi-Sichani, N.J. Moerke, M. Niepel, T. Zhang, N.S. Gray, P.K. Sorger, Systematic analysis of BRAF(V600E) melanomas reveals a role for JNK/c-Jun pathway in adaptive resistance to drug-induced apoptosis, Mol Syst Biol 11(3) (2015) 797.

[6] A. Wolf-Yadlin, S. Hautaniemi, D.A. Lauffenburger, F.M. White, Multiple reaction monitoring for robust quantitative proteomic analysis of cellular signaling networks, Proc Natl Acad Sci U S A 104(14) (2007) 5860-5.

[7] P.V. Hornbeck, B. Zhang, B. Murray, J.M. Kornhauser, V. Latham, E. Skrzypek, PhosphoSitePlus, 2014: mutations, PTMs and recalibrations, Nucleic Acids Res 43(Database issue) (2015) D512-20.

[8] D. Iglesias-Gato, P. Wikstrom, S. Tyanova, C. Lavallee, E. Thysell, J. Carlsson, C. Hagglof, J. Cox, O. Andren, P. Stattin, L. Egevad, A. Widmark, A. Bjartell, C.C. Collins, A. Bergh, T. Geiger, M. Mann, A. Flores-Morales, The Proteome of Primary Prostate Cancer, Eur Urol (2015).

[9] M. Niepel, M. Hafner, E.A. Pace, M. Chung, D.H. Chai, L. Zhou, B. Schoeberl, P.K. Sorger, Profiles of Basal and stimulated receptor signaling networks predict drug response in breast cancer lines, Sci Signal 6(294) (2013) ra84.

[10] J.V. Olsen, B. Blagoev, F. Gnad, B. Macek, C. Kumar, P. Mortensen, M. Mann, Global, in vivo, and site-specific phosphorylation dynamics in signaling networks, Cell 127(3) (2006) 635-48.

[11] F. Gnad, J. Gunawardena, M. Mann, PHOSIDA 2011: the posttranslational modification database, Nucleic Acids Res 39(Database issue) (2011) D253-60.

[12] F. Gnad, S. Ren, J. Cox, J.V. Olsen, B. Macek, M. Oroshi, M. Mann, PHOSIDA (phosphorylation site database): management, structural and evolutionary investigation, and prediction of phosphosites, Genome Biol 8(11) (2007) R250.
